# *Plasmodium vivax* infection-driven modulation of sterol carrier protein reveals a metabolic link to reproductive physiology in *Anopheles stephensi*

**DOI:** 10.3389/fimmu.2025.1703479

**Published:** 2025-12-11

**Authors:** Seena Kumari, Pooja Yadav, Nirmala Sankhala, Jyoti Rani, Gunjan Sharma, Charu Chauhan, Sanjay Tevatiya, Rajnikant Dixit

**Affiliations:** 1 Laboratory of Host-Parasite Interaction Studies, Department of Vector Genomics, Indian Council of Medical Research-National Institute of Malaria Research, New Delhi, India; 2 Academy of Scientific and Innovative Research (AcSIR), Ghaziabad, Uttar Pradesh, India

**Keywords:** mosquito, sterol-carrier protein, nutritional physiology, anti-*Plasmodium* immunity, reproduction

## Abstract

Mosquitoes rely exclusively on blood-derived sterols, transported by sterol carrier protein (SCP), for reproduction and metabolic regulation. SCP expression is strongly induced after blood feeding and dynamically modulated during *Plasmodium vivax* infection—upregulated in the midgut and salivary glands but suppressed in hemocytes—indicating that parasite infection modulates the host sterol transport and metabolism. RNAi-mediated knockdown of SCP significantly reduced mosquito fecundity, underscoring its vital role in reproductive physiology. These findings highlight SCP as a key metabolic regulator linking nutrient uptake to reproductive outcome, while its *Plasmodium* infection-driven modulation may facilitate the parasite growth. While reproduction remains the primary physiological outcome, the metabolic modulation of SCP during infection also points to it as a possible transmission-blocking target in integrated malaria control strategies.

## Introduction

Mosquitoes transmit numerous arboviral and parasitic diseases, including malaria, dengue, chikungunya, and Zika. Malaria is caused by *Plasmodium* species and transmitted by *Anopheles* mosquito species, and it remains a major global health and economic concern (1). Even though *Plasmodium falciparum* is the major cause of global morbidity, *Plasmodium vivax* is also a rapidly emerging problem in Southeast Asian regions. In India, highly endemic areas are facing an increased burden of both species, and thus, in parallel, there is an urgent need to focus on *P. vivax* (2). Blood feeding by adult female mosquitoes is vital for their egg development and gonotrophic cycle maintenance (3, 4). Following the ingestion of protein-rich blood meal, the gut metabolic machinery activates to digest the blood meal; to facilitate the conversion of blood to a nutritional supplement, the nutrients are supplied to the ovary for egg development *via* the fat body.

Cholesterol is a crucial biomolecule for maintaining the structural integrity of cell membranes, cell signaling, and larval metamorphosis. Kim et al. and Perera and Wijerathna demonstrated that an inhibitor targeting sterol carrier protein has shown larvicidal activity, highlighting the essential role of sterol carrier protein (SCP) in mosquito aquatic development, serving as a good candidate for a metabolic target for vector control (5, 6).

Moreover, it is the precursor for the ecdysone hormone responsible for vitellogenesis in adult female mosquitoes (7, 8). In vertebrates, SCP-2 is characterized as a lipid carrier protein, having a sterol binding domain (SCP-2, SCP-x, SCP-like-2, SCP-like-3, 17β-hydroxysteroid dehydrogenase type IV, and stomatin) that binds with sterols, fatty acids, fatty acid acyl-CoA, and phospholipids (9). Although the molecular mechanisms and fundamental nature of SCP-2’s ligand selectivity are ambiguous, it may mediate cholesterol trafficking, metabolism, and fatty acid uptake and trafficking (5, 10). Mosquitoes lack enzyme squalene monooxygenase and lanosterol synthase, which help in cholesterol *de novo* synthesis; hence, mosquitoes take a blood meal for cholesterol (6). Due to the hydrophobic nature of cholesterol, it cannot traverse the intracellular environment; thus, it needs SCPs to facilitate its transfer through the membrane (11). As cholesterol crosses the membrane, lipophorin assists its transport in hemolymph to the fat body and ovary for storage and egg development, respectively.

Although previous studies have demonstrated that the inhibition of SCP disrupts cholesterol uptake and results in larval mortality, the physiological role of SCP in adult mosquitoes remains poorly understood. Limited evidence suggests that *P. falciparum* and *Plasmodium berghei* infections can alter mosquito anti-parasitic immune responses; however, the potential involvement of SCP in maintaining nutritional homeostasis and cholesterol utilization during infection is yet to be elucidated (12). In the case of *P. vivax*, such investigations have been particularly constrained by the absence of a reliable and reproducible long-term *in vitro* culture system, which hampers mechanistic studies of parasite–vector interactions. Our recent comparative gut-RNA-Seq data analysis highlighted that *P. vivax* infection induces several unique transcripts that encode proteins such as trehalase, folliculin, and sterol carrier (13). These proteins may have a potential role in the management of nutritional homeostasis. A functional knockdown of trehalase enzyme transcripts led to a significant loss of egg development. However, *P. vivax* infection causes an increased expression of these transcripts in the gut and salivary glands of the *Anopheles stephensi* mosquitoes (14). Here, we aimed to evaluate the spatial–temporal expression of SCP in response to i) blood meal digestion, along with cholesterol absorption and its distribution in various tissues, and ii) *P. vivax* infection in the midgut, hemocyte, and salivary glands. We also aimed to evaluate the possible role in the reproductive physiology of the adult female *An. stephensi* mosquitoes. Here, we demonstrate that sterol carrier protein transcript (*AsSCP*) upregulates after a few hours in response to blood feeding in uninfected mosquitoes, while *P. vivax* infection boosts sterol carrier protein transcription in the gut and salivary glands. Our data suggest that blood meal-induced modulation of SCP favors optimal maturation of ovarian development. However, *P. vivax* infection may alter the nutritional stress response and influence anti-*Plasmodium* immunity in the mosquito. A significant reduction in the eggs laid by *AsSCP*-silenced gravid females correlates with its possible role in mosquito reproductive physiology.

## Materials and methods

### Mosquito collection, rearing, and maintenance

*An. stephensi* mosquitoes, an Indian strain, were taken from an established colony perpetuated in the insectary at ICMR-National Institute of Malaria Research and maintained under conditions of 28°C ± 2°C, ~80% relative humidity, and a photoperiod of 12:12 hr. Throughout the project, adult mosquitoes were regularly supplemented with sterile sugar solution (10%) using a cotton swab (15), while for maintenance initiation of gonotrophic cycles, 3–4-day-old mosquitoes were allowed to feed on a rabbit obtained from an institutional animal house facility. After 72 hrs of blood feeding, adult female mosquitoes were allowed to lay eggs on moistened filter paper pasted along the wall of a small plastic cup, one-third of which was filled with pre-cooled boiled water. Then, hatched larvae were transferred to enamel trays containing water and supplemented with mixed dried powder of fish food and dog biscuit in a ratio of 4:6 as larval feed. At a regular interval of 24 hrs, waste was removed, and a fresh nutrient supply to the larvae was provided. Following completion of larval stages (L1–L4), the mature pupae were manually collected in a water-containing plastic cup and kept inside a 30 × 30 × 30 cm^3^ muslin cloth cage for adult emergence. Post-emergence, the adult mosquitoes fed on soaked raisins and sterile water swabs. All the procedures for the rearing and maintenance of mosquito culture protocols were approved by the Institute Animal Ethics Committee (NIMR/IAEC/2017-1/07).

### Mosquito tissue sample collection

As per the technical design of the experiments under distinct physiological conditions, 3–4-day-old, naïve, sugar-fed, adult female *An. stephensi* mosquitoes were cold (4°C)-anesthetized for 5 min and immobilized in a drop of ice-cold Phosphate Buffered Saline (PBS) on microscopic slides. The desired tissues, such as midgut, salivary glands, hemolymph, fat body, and ovary, were dissected under a microscope and directly collected in a sterile 1.5-mL Eppendorf tube containing ~100 μL TRIzol as described earlier (16). Likewise, the developmental stages of the *An. stephensi* mosquitoes were monitored; egg, larvae (stages I–IV), and pupae were also collected in TRIzol reagent after removal of excess water using filter paper, and all the collected samples were stored at −20°C until RNA extraction. For hemolymph collection, approximately 2–3 μL of anticoagulant consisting of 60% Schneider’s medium, 10% fetal bovine serum, and 30% citrate buffer was injected into the thorax. The mosquitoes’ belly bulged out, and a small incision was made in the abdomen using a microscopic needle so that transparent hemolymph oozed out. Hemolymph was collected using a micropipette and pooled in TRIzol. The fat body was collected by pulling all the abdominal tissues from the last two abdominal segments, and afterward, force tapping of the abdominal carcass was conducted so that the fat body (pale yellow in color) oozed out (16).

### Infectivity assay

The blood sample was collected from the *P. vivax*-infected patients with the approval of the Ethics Committee of the National Institute of Malaria Research (NIMR), Delhi (ECR/NIMR/EC/2012/41). Written informed consent (IC) was obtained from donors visiting the NIMR clinic, and 0.5 to 2 mL of venous blood was drawn into heparin-containing tubes and kept at 37°C until feeding. For mosquito infection, overnight-starved, 4–5-day-old female *An. stephensi* mosquitoes were fed using a pre-optimized artificial membrane feeding assay (AMFA). The control mosquito group, originating from the same cohort, was offered uninfected blood obtained from a volunteer donor. After successful blood feeding, the unfed and partially fed mosquitoes were carefully removed, while fully fed mosquitoes were maintained under optimal insectarium conditions. The positive infection was confirmed via standard mercurochrome staining of the gut oocysts readily observed under a compound microscope, and the desired tissue samples, such as midgut, salivary glands, and hemocytes, were collected from 20–25 mosquitoes for subsequent analysis as described earlier (17).

### RNA extraction, cDNA synthesis, and gene expression analysis

Total RNA was extracted from the midgut, salivary glands, and other tissues following the standard manufacturer’s protocol involving phenol-chloroform extraction. The isolated total RNA was quantified by NanoDrop (ND-2000; Thermo Fisher, USA, Waltham, Massachusetts, USA), and each RNA sample was treated with RNase-free DNase I to exclude any genomic DNA contamination. One microgram of total RNA was utilized for the synthesis of first-strand cDNA using a mixture of oligo-dT and random hexamer primers, and PrimeScript II reverse transcriptase as per the described protocol (Verso cDNA synthesis Kit, Cat#AB-1453/A, EU, Lithuania) (15, 18).

Routine RT-PCR was performed using SCP-2 primer sequences Fw: AGTTGAAGGTGGAGAAAGGT and Rev: TGATTTACTGACGTACTGCG and housekeeping gene Actin primer sequences Fw: TGCGTGACATCAAGGAGAAG and Rev: GATTCCATACCCAGGAACGA and validated via agarose gel electrophoresis, while relative gene expression analysis was performed using Real-Time PCR. Approximately 25 ng of cDNA was used in 10-μL real-time PCR reactions. The relative abundance of the gene of interest was assessed using the SYBR Green qPCR master mix (Thermo Scientific) and the Bio-Rad CFX96 PCR machine. PCR cycle parameters involved an initial denaturation at 95°C for 15 min, and 40 cycles of 10 s at 95°C, 15 s at 52°C, and 22 s at 72°C. After the final extension step, the melting curve was derived and examined for quality control. Each experiment was performed in three independent biological replicates.

### Gene knockdown RNAi assays in adult mosquitoes

To knock down SCP expression, dsRNA primers carrying a T7 overhang were synthesized as listed in the **Supplementary Table (ST1)**. The amplified PCR product was examined via agarose gel electrophoresis, purified (Thermo Scientific Gene JET PCR Purification Kit #K0701), quantified, and subjected to double-stranded RNA synthesis using Transcript Aid T7 high-yield transcription kit (Cat# K044, Ambion, USA, Waltham, Massachusetts, USA), and the dsrLacZ gene was used as a control. Approximately 69 nL (3 μg/μL) of purified dsRNA product was injected into the thorax of a cold-anesthetized, 1–2-day-old female mosquito using a nano-injector (Drummond Scientific, CA, USA, Broomall, Pennsylvania, United States), as described earlier (14, 16). The knockdown of the respective gene was confirmed via quantitative RT-PCR after 3–4 days of dsRNA injection.

### Oviposition assay

Overnight, 50 mated female (either control or SCP knockdown) mosquitoes were blood-fed on a rabbit. After blood feeding, partially/incompletely blood-fed females were removed; fully engorged females were kept in a cage for 48 hrs and then individually placed in netted plastic cups containing half-filled water and paper fixed on the wall of cups, which provided a moisture surface to lay eggs. After 24 hrs, the total number of eggs laid on the blotting paper was manually counted and compared between the two mosquito groups. The number of eggs laid was recorded in three independent experiments.

### Statistical analysis

The relative quantification data of the gene expression were normalized with an internal control (Actin) and analyzed using the 2^−ΔΔCt^ method in the Origin8.1 software. Initially, relative expression was evaluated as a general response using one-way analysis of variance (ANOVA) through multiple comparisons. However, wherever required, “test” sample data were compared with “control” data set and statistically analyzed using Student’s t-test. All the data were expressed as mean ± SD, and the results were significant if the *p*-value was less than 0.05. Each experiment was performed at least three times to validate the findings. Egg-laying statistical analysis using an unpaired two-tailed Student’s t-test, with *p* < 0.05, confirmed the statistical significance of the reduction.

### Gene phylogeny analysis

Standard BLAST homology search analysis was performed to retrieve the corresponding homolog sequences from the vector base. Phylogenetic trees were prepared with selected SCP amino acid sequences using the maximum likelihood (ML) method in the MEGA X program.

All our selected SCP sequences were aligned using the ClustalW algorithm, where the reliability of the branching was tested using a bootstrap analysis with 1,000 replicates. The processed phylogenetic tree was examined based on clusters and nodes formed.

## Result and discussion

### Identification and molecular characterization of *AsSCP*

To unravel the tissue-specific molecular complexity of mosquito–parasite interaction, currently, we are annotating a large-scale RNA-Seq database generated from naïve control versus *P. vivax*-infected mosquitoes (13). *Plasmodium* invades different tissues such as the midgut, hemolymph, and salivary glands. These tissues are subjected to various immune responses and altered metabolic conditions. The successful development of *Plasmodium* heavily depends upon the host’s nutrient resources. Parasites sense the host environment and respond quickly to metabolic fluctuations to survive within their host. Thus, identification of the host factors implicated in the host–parasite interaction is crucial in comprehending the growth and development of the parasite within the vector and uncovering novel approaches to intervene in *Plasmodium* development. While studying how *Plasmodium* development within the vector modulates the nutritional homeostasis of the host to favor its development, we observed an increased read count of a transcript encoding sterol carrier homolog protein (which facilitates the intracellular transport of cholesterol/lipid) in *P. vivax*-infected mosquitoes (13).

**Figure 1:**
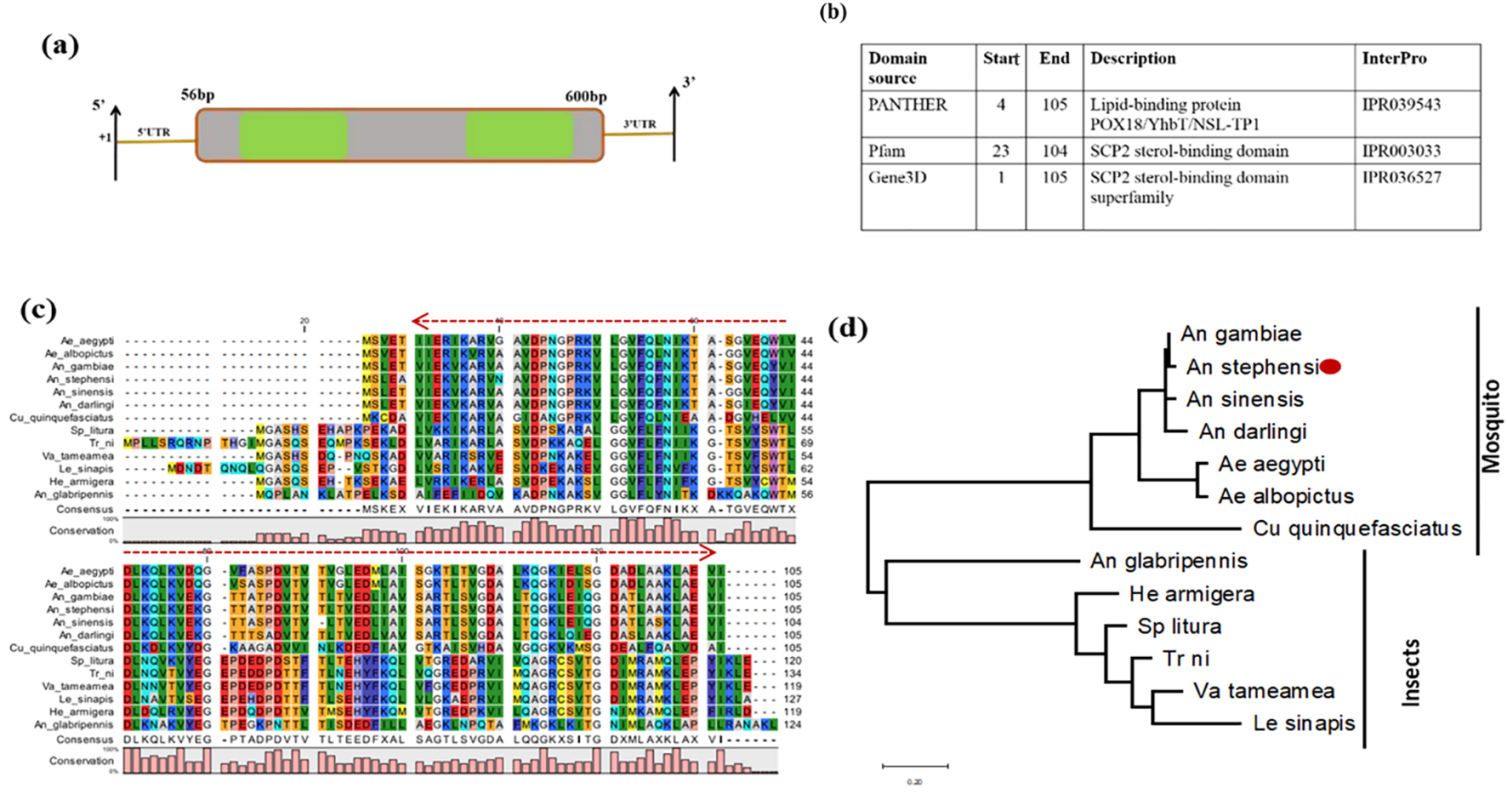
Genomic organization and molecular characterization of *AsSCP*. **(a)** Schematic representation of the genomic architecture of SCP. Two green-colored boxes (E1 and E2) represent exons, and +1 indicates the transcription initiation site. A 50-bp UTR region is present on both 5′ and 3′ ends of the transcript. **(b)** SCP predicts domain description. **(c)** Multiple sequence alignment of mosquitoes and other insect-encoded SCP homolog protein showing strong conservation among anopheline mosquitoes; red arrow highlights the conserved lipid-binding domain. **(d)** Phylogenetic relationship of *AsSCP* proteins within the insect taxa species: non-blood-feeding insects included *Anoplophora glabripennis*, *Spodoptera litura*, *Helicoverpa armigera*, *Vanessa tameamea*, *Trichoplusia ni*, and *Leptidea sinapis*, while blood-feeding insects included mosquito species of *Aedes aegypti*, *Aedes albopictus*, *Culex quinquefasciatus*, *Anopheles darlingi*, *Anopheles sinensis*, *Anopheles gambiae*, and *Anopheles stephensi*. The evolutionary history was built using the maximum likelihood method and Jones-Taylor (JT) matrix-based model involving 13 amino acid sequences. The heuristic search was obtained by applying neighbor-joining and BioNJ algorithms to a matrix of pairwise distances estimated, followed by topology selection with the superior log-likelihood value. The tree is drawn to scale, with branch lengths measured in the number of substitutions per site. SCP, sterol carrier protein.

Like other insects, mosquitoes cannot synthesize *de novo* cholesterol and must acquire it from a blood meal. We further characterized and evaluated the SCP expression in the mosquito to examine its possible physiological role in the mosquito. An *in silico* analysis revealed that *AsSCP* (*ASTEI04293-PA*) is a 655-bp-long transcript, encoding 105 amino acids, and has a high degree of conservation among the insects and blood-feeding mosquito species (**Figures 1a–d**). *AsSCP* comprises two exons followed by 3′ and 5′ UTR regions, but the putative conserved domain is still obscure. However, a comprehensive search of the mosquito-specific genomic database suggested that *AsSCP* may carry a poorly characterized “lipid-binding protein POX18/YhbT/NSL-TP1” domain. Non-specific lipid-transfer protein-like 1 (NSL-TP1 or nlt-1) from *Caenorhabditis elegans* is found in the peroxisome (19), while protein YhbT from *Escherichia coli* is cytosolic. POX18 from the yeast *Candida tropicalis* is involved in beta-oxidation of long-chain fatty acids and non-specific lipid-transfer activity, despite lacking the catalytic cysteine conserved in SCP2.

### Developmental expression pattern of *AsSCP* in *An. stephensi*

Although we observed a high expression of *AsSCP* in the pupal stage, after emergence, the adult male mosquitoes exhibited higher expression compared to the adult female mosquitoes (**Supplementary Figure 1**). The transformation from pupa to adult is brought about by ecdysteroid and a decline in the level of juvenile hormone (JH) during the pupal stage (20). Together, these data indicate that the active pupal stage may serve as a reserve source of cholesterol mobilization for the synthesis of ecdysone during the final molting of pupa to adult mosquitoes. Although the exact mechanism is yet to be elucidated, the knockdown of SlSCPx transcripts in the *Spodoptera litura* larvae adversely affected the transition from the larval to pupal stage (21).

The altered cholesterol accumulation in response to SlSCPx knockdown suggests that *AsSCP* may have a similar effect. Likewise, SCP inhibitors suppressed cholesterol uptake in *Aedes aegypti* fourth instars and showed larvicidal activities (22).

### Tissue-specific and blood meal-induced expression of *AsSCP* in *An. stephensi*

**Figure 2:**
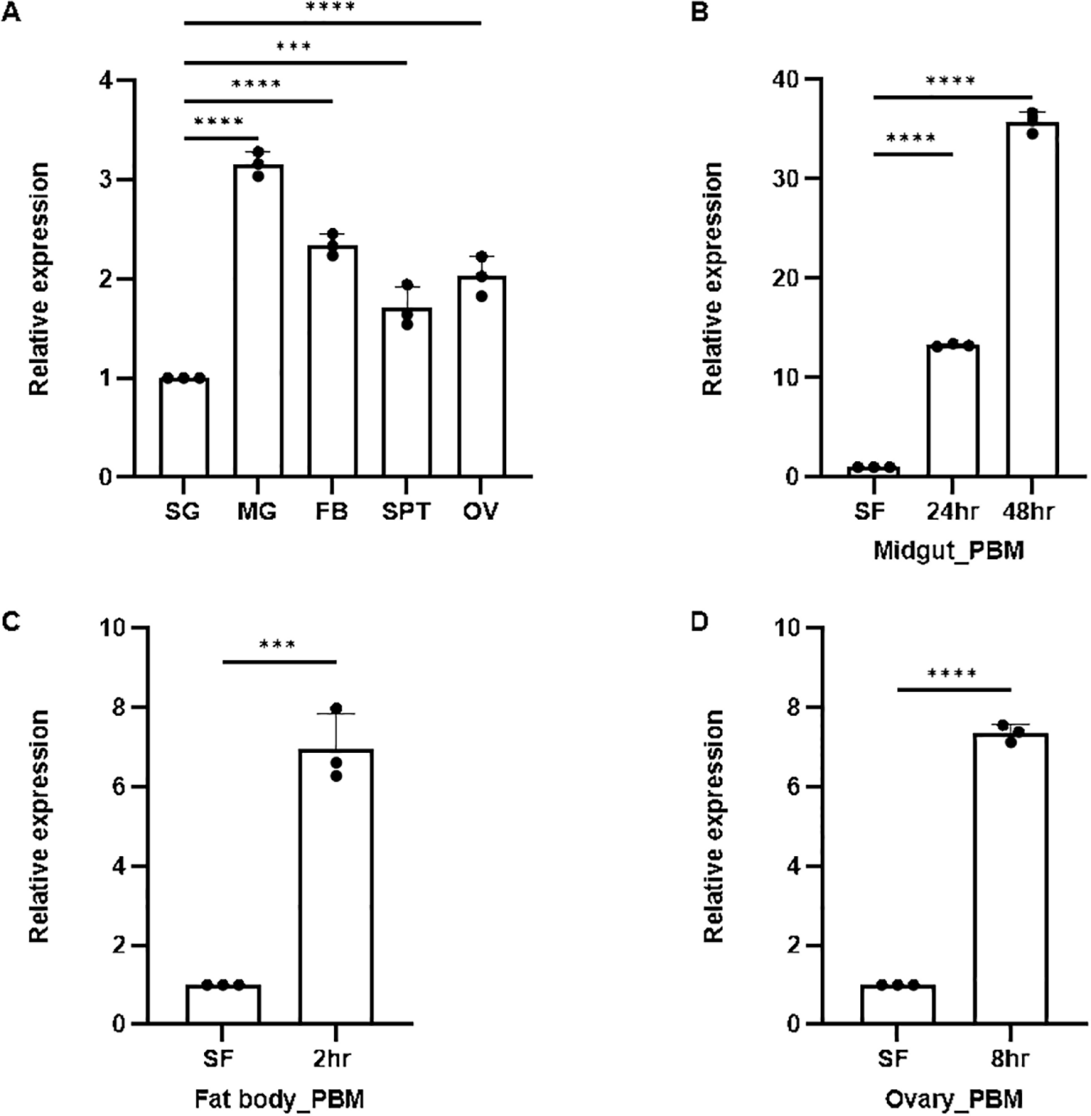
Spatio-temporal expression of SCP. **(A)** Real-time PCR-based tissue-specific expression analysis of SCP in different tissues of female mosquitoes observed highest expression in MG (*p* < 0.0001), FB (*p* < 0.0001), OV (*p* < 0.0001), and SPT (*p* < 0.0006). Here, salivary gland was considered as control for each test sample. **(B)** Relative expression profiling of SCP in the midgut after blood meal (24 hrs PBM and 48 hrs): SF (sugar-fed) was considered as control. All the samples were collected in three biological replicates and analyzed with the help of one-way ANOVA (Dunnett’s multiple comparison test) in GraphPad Prism. **(C)** Relative expression profiling of SCP in the FB after 2 hrs (*p* < 0.0003) blood meal: SF (sugar-fed) FB was considered as the control. **(D)** Relative expression profiling of SCP in the ovary after 8 hrs (*p* < 0.0001) blood meal: SF (sugar-fed) OV was considered as the control. Three independent biological replicates (n = 3, N = 30) were considered for statistical significance (*p < 0.05, **p < 0.005, ***p < 0.0005 and ****p < 0.0001) and calculated using unpaired two-tailed Student’s t-test with the help of GraphPad Prism. n, number of replicates; N, the number of mosquitoes pooled for sample collection; PBM, post blood meal; BF, blood fed; SG, salivary gland; MG, midgut; SPT, spermatheca; FB, fat body; OV, ovary; SF, sugar-fed; SCP, sterol carrier protein.

An enriched expression of *AsSCP* in the gut compared to other tissues in the naïve adult female mosquitoes (**Figure 2a**) prompted us to evaluate the effect of blood meal on its expression. First, we profiled and compared detailed time-dependent transcriptional responses of *AsSCP* in the midgut of blood-fed mosquitoes. Since blood meal serves as an excellent nutritional source through vitellogenesis and engages multiple organs, including the fat body and ovaries in storage and mobilization, we also monitored *AsSCP* expression in the fat body and the ovary of gravid female mosquitoes. Our observation of the gradual induction of *AsSCP* within the midgut epithelium (**Figure 2b**; also see **Supplementary Figure 2**) supports the idea that *AsSCP* may favor the absorption of cholesterol in the gut lumen (23). Our results are consistent with earlier work in *Anopheles sacharovi*, where SCP-2 transcripts were found to be highly enriched in the midgut and fat body. Similar expression patterns were observed in *An. stephensi*—with *AsSCP* strongly expressed in both tissues and further upregulated following a blood meal—suggesting a conserved role for SCP in regulating lipid assimilation and metabolism across anopheline species (24).

Large amounts of required proteins, such as lipophorin, responsible for lipid/sterol transport in circulation, or vitellogenin for egg maturation, are secreted by the fat body (20). Furthermore, in parallel, we observed an early [2 hr post blood meal (PBM)] multi-fold induction of *AsSCP* in the fat body (*p* < 0.0003; **Figure 2c**, **Supplementary Figure 3**). The results indicate that *AsSCP* may be involved in the transportation of cholesterol/sterol from the fat body to the hemolymph side for loading into lipophorin, in due course incorporating into the developing oocytes. Previous literature supporting this notion states that 24 hrs post blood meal, sterol from the fat body is utilized in the developing oocytes of *Ae. aegypti* (22). In the case of the ovary, a high expression of *AsSCP* 8 hr PBM was observed (*p* < 0.0001; **Figure 2d**, **Supplementary Figure 4**).

### Modulation of *AsSCP* during *P. vivax* infection

Cholesterol is essential for cell membranes and cell signaling; therefore, its consumption by intracellular parasites for their development is typical in the host-vector. For example, *Toxoplasma gondii* acquires cholesterol from the host cells, and any interference with cholesterol uptake by the parasite significantly reduces the viability of the parasite (25). In mammalian hosts, the *Plasmodium* parasite scavenges the cholesterol lipid from the host erythrocyte membrane (26). Likewise, the *Plasmodium* oocysts in mosquito vectors may acquire fatty acids and phospholipids for developing oocysts and their structural development *via* lipophorin (LP), although during its development within the mosquito host, *Plasmodium* elicits tissue-specific differential immuno-physiological responses (27). However, the exact nature of the SCP involvement in *Plasmodium* infection is yet unclear.

**Figure 3:**
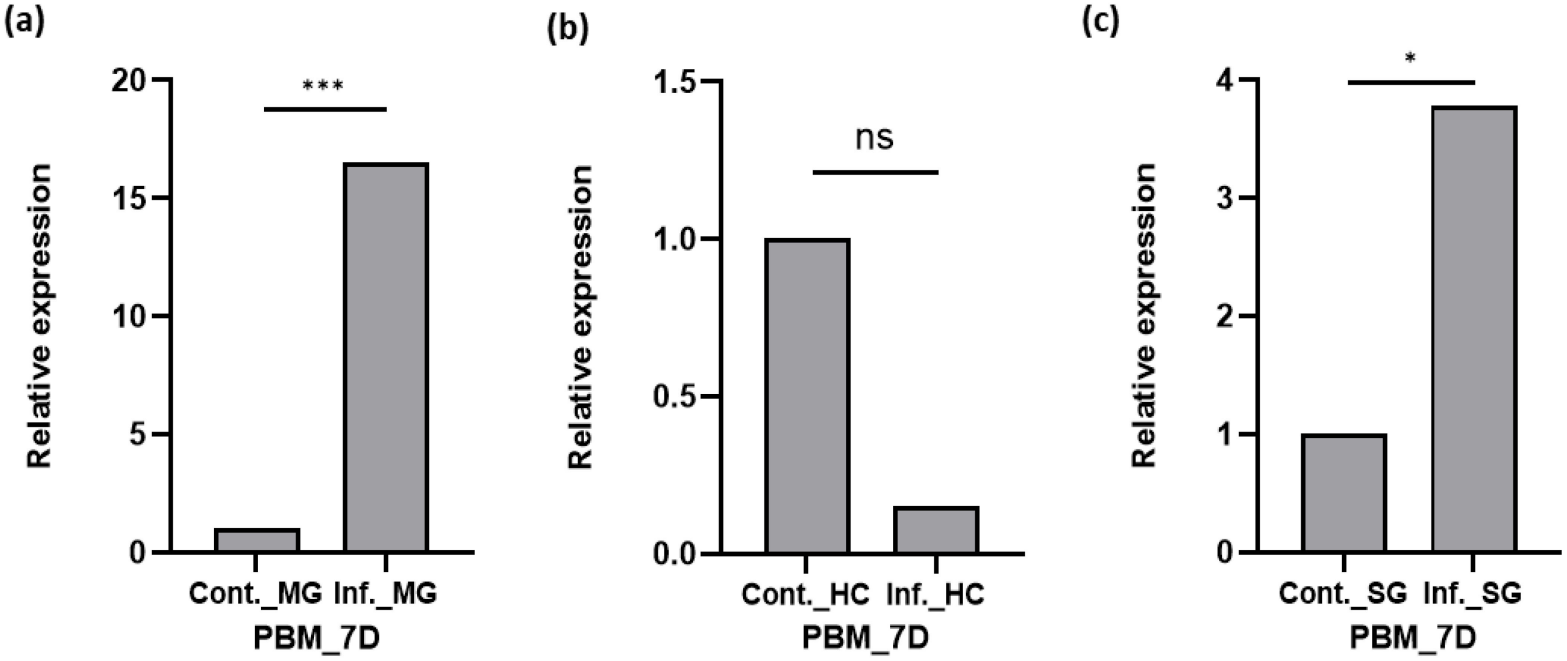
Transcriptional profiling of SCP in different tissues after *Plasmodium vivax* infection of mosquitoes. **(a)** Relative expression profiling of SCP in the midgut after *P. vivax* infection time series and control normal blood meal midgut (MG). **(b)** Relative expression profiling of SCP in hemocyte **(HC)** after *P. vivax* infection and control normal blood meal. **(c)** Relative expression profiling of SCP in salivary gland (SG) after *P. vivax* infection and control normal blood meal SG. Three independent biological replicates (n = 3, N = 30) were considered for statistical significance (*p < 0.05; **p < 0.005, and ***p < 0.0005) and calculated using Student’s t-test. n, the number of mosquitoes pooled for sample collection; N, number of replicates; HC, hemocyte; SG, salivary gland; PV, *P. vivax*; C, control; INF, infected; D, day; BF, blood-fed; SCP, sterol carrier protein. ns, non-significant.

Because *Plasmodium* is incapable of synthesizing *de novo* cholesterol and heavily depends on the host’s nutritional resources for its development, therefore, we investigated whether the *P. vivax* infection alters the expression of SCP in the midgut (site for oocyst for 6–8 days), hemocyte (an immune organ), and salivary glands (hosts and stores sporozoites for *Plasmodium* transmission). We discerned that *Plasmodium* infection (**Figure 3**) induces a multifold SCP expression in the midgut 7 days post-infection (DPI; *p* < 0.000935; **Figure 3a**; also see **Supplementary Figure 5**), but hemocyte tissues depicted suppression of *AsSCP* expression (**Figure 3b**), while again, a heightened expression in the salivary glands was readily observable in the salivary glands (*p* < 0.011435; **Figure 3c**). Taken together, these observations suggest that the pronounced enrichment of SCP in the midgut and salivary glands may contribute to the provision of lipids or sterols to developing oocysts and emerging sporozoites, respectively. Our findings are consistent with previous reports showing that *P. falciparum* sporozoites significantly deplete lipid reserves in *Anopheles gambiae*, underscoring the importance of host-derived lipids for parasite development. In addition to a potential role in supplying cholesterol and other lipids to the oocyst, the modulation of AsSCP during *P. vivax* infection may reflect parasite-induced metabolic adjustments, reminiscent of the lipid metabolism remodeling described in the midgut of *Ae. aegypti* (28) during bacterial and viral infections. However, the lack of a reliable *in vitro* culture system for *P. vivax* limits our ability to directly assess the functional consequences of SCP modulation on parasite development and mosquito anti-*Plasmodium* immunity.

### Functional knockdown (RNA silencing) of *AsSCP* and impact on mosquito fecundity

An early induction of *AsSCP* in the fat body and ovary after blood feeding compelled us to check the impact of *AsSCP*’s role on the reproductive physiology of the uninfected female mosquito, as the fat body and ovary are central tissues to mosquito reproduction. The ovary utilizes cholesterol for the biosynthesis of ecdysone hormone to initiate the vitellogenesis process.

**Figure 4:**
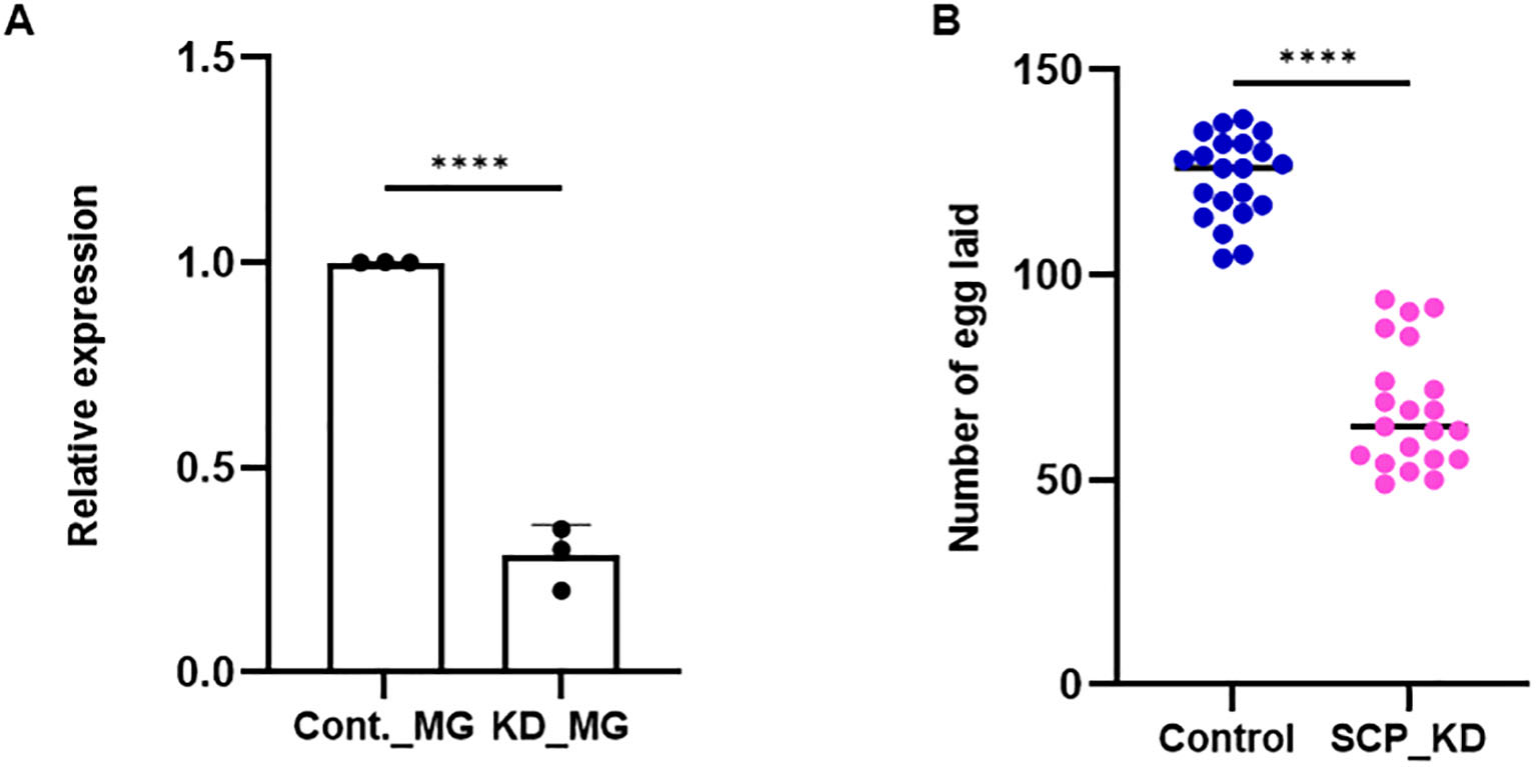
SCP silencing effect on fecundity. **(a)***AsSCP* silencing exhibited a >70% reduction in mRNA expression level as compared to control (*p* < 0.0001), as tested in the MG. A minimum of three biological replicates were performed and analyzed with the help of unpaired two-tailed Student’s t-test. **(b)** After mating with age-matched healthy male mosquitoes, *AsSCP*-silenced female mosquitoes were offered blood meal from rabbit, and 72 hrs after meal, silenced mosquitoes laid reduced number of eggs (*p* < 0.0001) compared with the control mosquito group. A minimum of three biological replicates were performed and analyzed with the help of unpaired two-tailed Student’s t-test (n = 3, N = 7 females/trial). n, number of replicates; N, the number of mosquitoes pooled for sample collection. All the statistical analyses were conducted with the help of GraphPad Prism. Cont., control; MG, midgut; KD, knockdown; SCP, sterol carrier protein. Statistically, **** represents data significance, which marks *p* < 0.0001.

The gene silencing strategy was followed, and an oviposition assay was performed. A significant depletion (~70%/*p* < 0.0001) of *AsSCP* mRNA was noticeable in the midgut (**Figure 4a**) of the adult female mosquitoes. After mating with naïve adult male mosquitoes, both silenced and control adult female mosquito groups were subjected to a blood meal to monitor reproductive success. A significant reduction (*p* < 0.0001) in the egg numbers laid by *AsSCP*-silenced adult female mosquitoes compared with controls (**Figure 4b**) suggests that SCP may have a direct role in the reproductive physiology of this mosquito species. These results reinforce SCP’s central role in reproductive physiology; however, at the same time, its nutritional role may represent a potential vulnerability to parasites, and thus, by impairing both fecundity and parasite-supportive pathways, SCP provides a dual advantage that may also inform future vaccine-oriented interventions.

In summary, previous studies have shown that SCP is essential for mosquito development, as chemical inhibition disrupts lipid uptake and causes larval mortality. Our findings extend this role to adults, demonstrating that RNAi-mediated suppression of AsSCP markedly reduces fecundity, underscoring SCP’s importance in both larval survival and adult reproductive success.

## Conclusion

**Figure 5:**
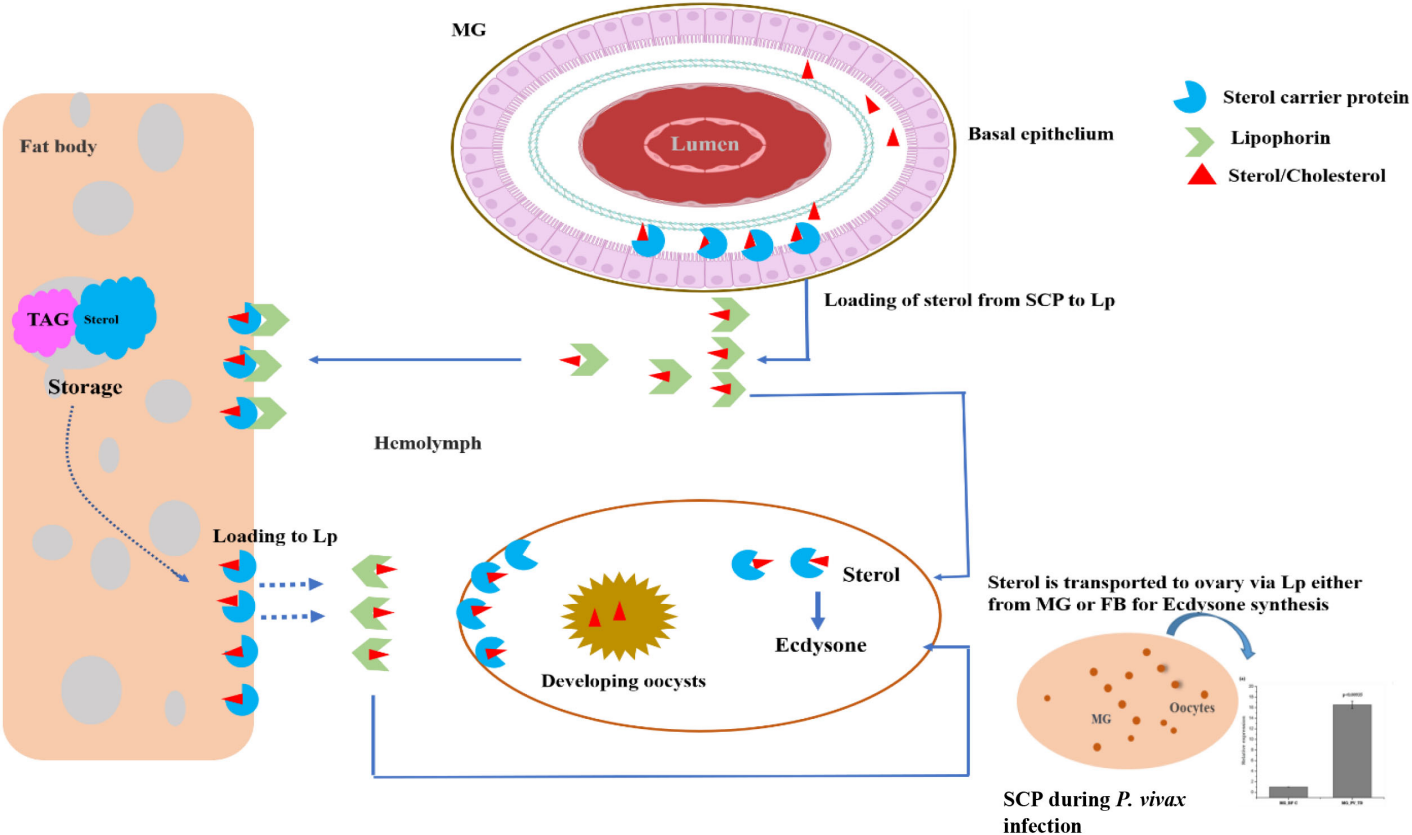
A proposed hypothesis for the possible role of *AsSCP* in the adult female mosquito. The mosquito acquires the dietary cholesterol from the blood meal once inside the midgut epithelial cell. A small fraction of sterol, along with other lipids, is stored in the lipid droplet of the midgut, and the remaining amount of sterol/lipid is transported intracellularly toward the basal side of the midgut *via* SCP and loaded into lipophorin for transportation to the fat body. During oogenesis, SCP facilitates the mobilization and transport of stored sterol within the fat body and loads it into lipophorin for the developing oocyte. During the early hours of blood feeding, the sterol is intracellularly transported to the site of utilization within the ovary, where sterol is converted into ecdysone to initiate the process of vitellogenesis. A blood meal or a fat body could be the source of sterol in the ovary. Although SCP induction after blood feeding suggests a link to ecdysone production via sterol transport, we did not measure ecdysone; LC–MS/MS-based quantification is needed to confirm this metabolic connection. SCP, sterol carrier protein.

SCP inhibitors are known to exhibit larvicidal properties. However, its role in the regulation of nutritional physiology and *Plasmodium* development remains uncertain. In our study, we characterized and evaluated the molecular responses of the sterol carrier protein (*AsSCP*) gene in response to blood feeding and *Plasmodium* infection in the *An. stephensi* mosquitoes. Although we do not provide any functional experimental proof, our transcriptional profiling concerning *P. vivax* infection suggests that *AsSCP* may be providing lipids/sterols to the developing oocysts. However, further additional studies based on SCP’s knockout or knockdown infection assay will clarify its specific role in *Plasmodium* growth and survival. Tissue-specific alteration in response to blood meal uptake and functional knockdown experiments demonstrated that *AsSCP* probably plays a crucial role in egg development. While reproduction remains the dominant phenotype, its dual involvement in nutrition and infection suggests that SCP may serve as a bridge between physiology and immunity. Here, we propose that *AsSCP* (**Figure 5**) may serve as a unique target to disrupt nutrition-dependent *Plasmodium* development as well as mosquitoes’ reproduction, opening the door for innovative transmission-blocking tools for malaria control.

## Limitations and future directions

The findings of our study demonstrate that SCP is associated with mosquito reproduction and modulation in its gene expression pattern during *P. vivax* infection, although we have provided no experimental evidence of how it influences ecdysone production or *Plasmodium* growth. Due to limitations of *P. vivax* culture and/or availability of *P. vivax*-infected blood samples, we were not able to check the parasite load after SCP silencing. In future studies, if possible, we intend to carry out an infection study in RNAi-silenced mosquitoes to check how the knockdown of SCP influences the parasite growth and transmission if we are able to obtain fresh infected samples. Additionally, we also intend to assess the hormone level using Liquid Chromatography Coupled with Tandem Mass Spectrometry (LC–MS/MS) in SCP-silenced female mosquitoes after blood feeding to check whether impaired sterol transport results in lower ecdysone production and egg formation. This functional experiment will clarify SCP’s role in mosquito metabolism, reproduction, and infection.

## Data Availability

The SCP was identified from the RNAseq data of the midgut and was deposited to NCBI with Accession number (Migut-control: SRR8580010; Midgut-Infected: SRR8580011).
