# Supplementary material for: Plasmodium vivax infection-driven modulation of sterol carrier protein reveals a metabolic link to reproductive physiology in Anopheles stephensi
Source: Front Immunol. 2025 Dec 11;16:1703479. doi: 10.3389/fimmu.2025.1703479 (PMC12738954; doi:10.3389/fimmu.2025.1703479)
Supplement: Supplementary file 1 [file DataSheet1.pdf]

Supplemental Data

Plasmodium vivax infection-driven modulation of Sterol Carrier Protein reveals a metabolic link to reproductive physiology in *Anopheles stephensi*

Seena Kumari[1$], Pooja Yadav[1$,2], Nirmala Sankhala[1,2], Jyoti Rani[1], Gunjan Sharma[1,2], Charu Chauhan[1], Sanjay Tevatiya[1*], Rajnikant Dixit[1,2]*

1. Laboratory of Host-Parasite Interaction Studies, Department of Vector Genomics, ICMR-National Institute of Malaria Research, Dwarka, New Delhi, 110077, India.
2. Academy of Scientific and Innovative Research (AcSIR), Ghaziabad, Uttar Pradesh-201002.

$Equal contribution

*Correspondence: sanjaycena51@gmail.com, rkdixit@icmr.gov.in

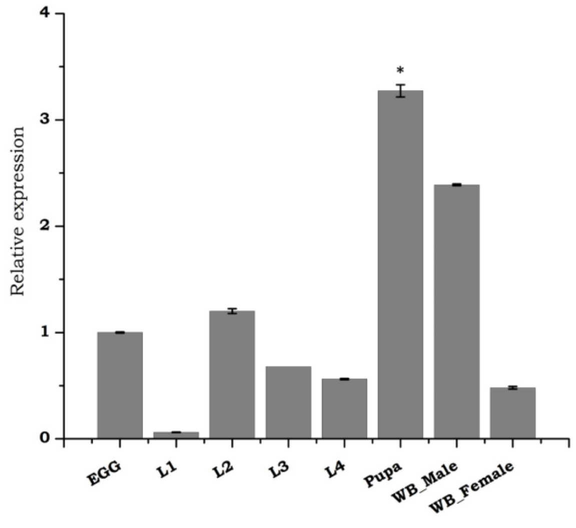

Figure 1. Relative expression profiling of SCP in different developmental stages: Egg, L1(larval 1), L2(larval 2), L3 (larval 3), L4 (larval 4), Pupa ($p<0.002401$), male, and female mosquito. Three independent biological replicates (n=30, N3) were considered for statistical significance $*p<0.05$; $**p<0.005$ and $***p<0.0005$ was calculated using Student's *t-test*. (*n*=represents the number of mosquito pooled for sample collection; *N*= number of replicates).

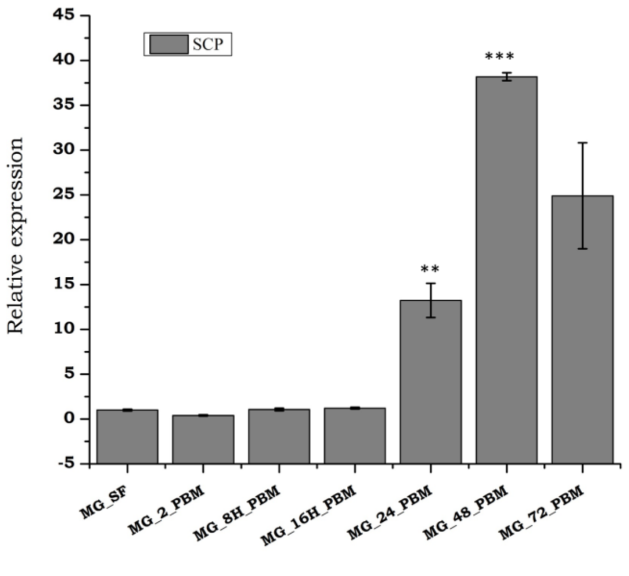

Figure 2. Relative expression profiling of SCP in the midgut after blood meal; 2hr,8hr,16hr,24hr, 48hr, and 72hr. Three independent biological replicates (n=30, N3) were considered for statistical significance $*p<0.05$; $**p<0.005$, and $***p<0.0005$ was calculated using Student's *t-test*. (*n*=represents the number of mosquito pooled for sample collection; *N*= number of replicates).

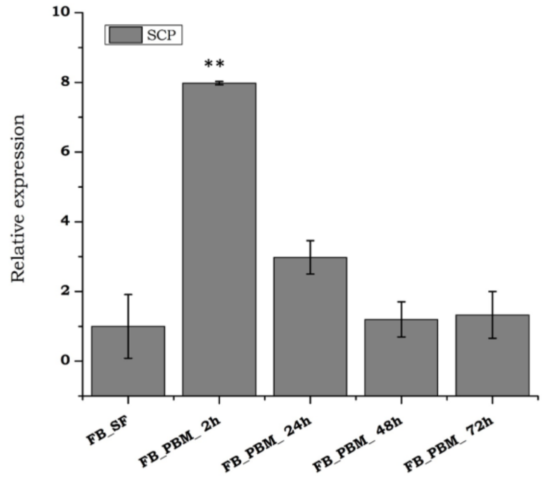

Figure 3. Relative expression profiling of SCP in the fat body after blood meal; 2hr, 24hr,48hr, and 72hr. Three independent biological replicates (n=30, N3) were considered for statistical significance *p<0.05; **p<0.005, and ***p<0.0005 was calculated using Student's *t-test*. (*n*=represents the number of mosquitoes pooled for sample collection; *N=number* of replicates)

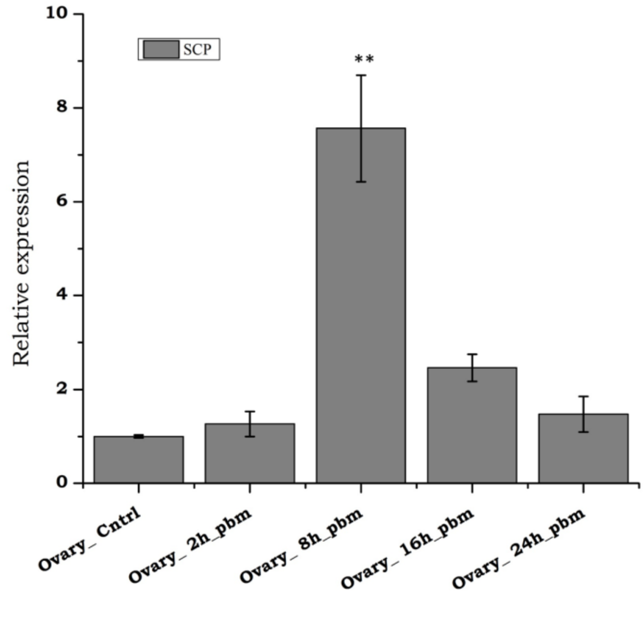

Figure 4. Relative expression profiling of SCP in the ovary after a blood meal; 2hr,8hr,16hr and 24hr. Three independent biological replicates (n=30, N3) were considered for statistical significance *p<0.05; **p<0.005, and ***p<0.0005 was calculated using Student's *t-test*. (*n*=represents the number of mosquito pooled for sample collection; *N*= number of replicates).

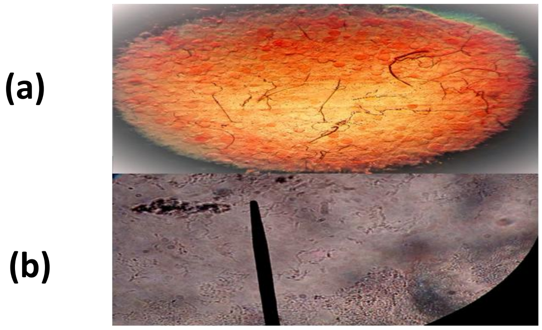

Figure 5: (a) Mosquito midgut shows super *Plasmodium* infection oocyte number > 250-300 **(b)** The mosquito salivary gland presents thousands of sporozoites

Table:1 Primer sequnces

S.No.	Gene Name	Primer sequences
1	Actin	Fw: TGCGTGACATCAAGGAGAAG<br>Rev: GATTCCATACCCAGGAACGA
2.	SCP_rt pcr	Fw: AGTTGAAGGTGGAGAAAGGT<br>Rev: TGATTTACTGACGTACTGCG
3.	SCP_DSR	Fw: TAATACGACTCACTATAGGGCTGTTATCGAGAAGGTGAAG<br>Rev: TAATACGACTCACTATAGGGCATCACCCTGAATCTCAA
